# Two fatal cases of plague after consumption of raw marmot organs

**DOI:** 10.1080/22221751.2020.1807412

**Published:** 2020-08-21

**Authors:** Jan Kehrmann, Walter Popp, Battumur Delgermaa, Damdin Otgonbayar, Tsagaan Gantumur, Jan Buer, Nyamdorj Tsogbadrakh

**Affiliations:** a Institute of Medical Microbiology, University Hospital Essen, University of Duisburg-Essen, Essen, Germany; b HyKoMed, Dortmund, Germany; c MeshHp, Essen, Germany; d National Center for Zoonotic Disease Ministry of Health, Ulaanbaatar, Mongolia; e MedClean LLC, Ulaanbaatar, Mongolia

**Keywords:** Plague, Mongolia, marmot, epidemiology, sepsis

## Abstract

Marmots are an important reservoir of *Yersinia pestis* and a source of human plague in Mongolia. We present two fatal cases of plague after consumption of raw marmot organs and discuss the distribution of natural foci of *Y. pestis* in Mongolia.

## Letter

Plague, caused by *Yersinia pestis,* is one of the most dreaded diseases in the world and has killed millions of people over the centuries [1]. *Y. pestis* has acquired virulence factors that make it a unique member of the family of *Yersiniaceae*, transmitted as a predominantly vector-borne pathogen. Plague is an endemic disease in many parts of the world. Wild rodents are an important reservoir of *Y. pestis* and its maintenance relies on flea vectors and climate [2]. Climate conditions affect all three components of the plague cycle: bacteria, vectors and animal hosts. Marmots are the main reservoir of *Y. pestis* in Mongolia and are an important source of human infection [3,4]. *Y. pestis* is commonly transmitted by fleabites. However, plague after the consumption of raw meat has rarely been reported [5-7]. Here, we present two fatal cases of plague caused by eating raw marmot organs, and we discuss the epidemiology of *Y. pestis* infection of small rodents in Mongolia.

A 38-year old Mongolian resident of Kazakh ethnicity from Bayan Ulgii aimag (province) in western Mongolia worked as a border guard. He called the emergency medical service from his home, reporting fever, abdominal pain, and bloody vomitus. Soon after his call, he died (28 April 2019). He had hunted and prepared marmots on 22 and 25 April and had been vaccinated with an EV76 *Y. pestis* vaccine one year previously. He and his 37-year old wife had consumed meat and raw marmot organs (kidney, stomach and gallbladder) on 22, 23, and 25 April. His wife visited a physician daily from 26 to 28 April because of fever, diarrhoea, abdominal pain, vomiting, and headache. She reported celebrating with friends between 22 and 25 April but concealed her contact with and consumption of raw marmot meat. Retrospective interviews with neighbours and older children revealed that the man and his wife were the only persons in the group of friends who had consumed raw marmot meat. The wife refused in-hospital diagnostic tests and was treated as outpatient with erythromycin and anti-inflammatory drugs. After the husband died at home, a tentative diagnosis of plague was made for the wife, and she was treated in the hospital with intravenous gentamicin and ceftriaxone. However, she died on 1 May. The couple left behind four children aged between nine months and twelve years.

Autopsy of the husband on 29 April found no fleabites or enlarged lymph nodes. His inner organs (stomach, oesophagus, liver, kidneys, and lungs) were enlarged and blood-filled and showed signs of inflammation. *Y. pestis* was cultured on Hottinger blood agar and detected by PCR with Pla1 (5’GAATGAAAATCTCTGAGG3’) and Pla2 (5’TCCAGCGTTAATTACGG3’) and pFRA1 (5’TCAGTTCCGTTATCGCC3’) and pFRA2 (5’GTTAGATACGGTTACGGT3’) primers from blood, liver, spleen, lung, kidney, stomach, brain and bone marrow. The identification was confirmed by phage lysis according to the Mongolian national guidelines. The wife presented with pharyngeal inflammation and swollen cervical lymph nodes. *Y. pestis* was detected by PCR and by culture of samples from an enlarged cervical lymph node. *Y. pestis* was also detected in swab specimens from the inflamed throat, blood, and gut.

Although both cases involved zoonotic and not interhuman infections, the detection of *Y. pestis* in the lungs during autopsy prompted the authorities to follow infection prevalence measures. Because it was possible that both patients had contracted secondary pneumonic plague, the authorities followed the Mongolian national plague guidelines for pneumonic plague. All 124 persons who had come into close contact with the couple between 22 and 28 April were treated prophylactically with doxycycline or ciprofloxacin. Ulgii city (34,000 residents) was quarantined from 1 to 6 May. F1-antigen tests were performed on throat swab specimens of 198 close contact persons, family members, friends, colleagues, and medical staff at least six days after the last contact, and all results were negative.

**Figure 1.**
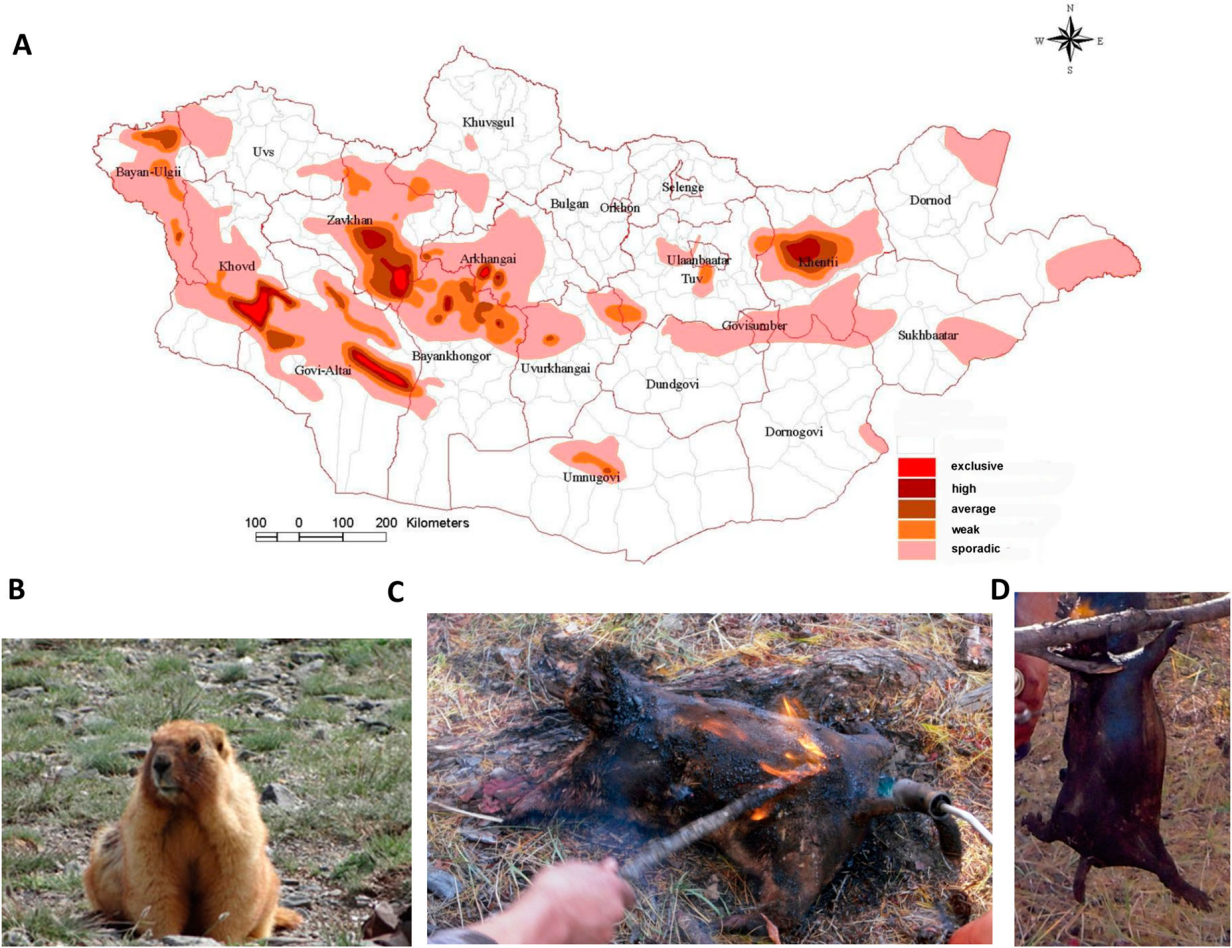
**A.** Geographic distribution of natural foci of plague in Mongolia as assessed from 2012 to 2019. Categorization of prevalence took into account the extent and continuity of *Y. pestis* infection of small rodents in various regions of Mongolia over the past eight years. For each aimag (province), a minimum of 80–100 small rodents and fleas associated with the rodents in an area 100–120 kilometres square were examined annually with serologic tests (F1-antigen and plague specific antibody test) and polymerase chain reaction (PCR). Positive samples were subjected to bacteriologic culture. **B.** Picture of *Marmota sibirica.* **C** and **D.** Preparation of traditional marmot boodog: Burning furs with flame (C) and cooked marmot boodog (D).

Annual surveys of the prevalence of *Y. pestis* in Mongolia from 2012 to 2019 found that 137 soums (districts) of 13 aimags have natural plague foci, with a high infection prevalence for Bayan Ulgii in the western part of the country (Figure 1(A)). The infection prevalence of marmots and their associated fleas in Bayan Ulgii aimag was 20.2% in 2018 and 16.8% in 2019. It was determined from testing 287 small rodents and 261 fleas in 2018 and 397 small rodents and 312 fleas in 2019 in this area by serologic tests and PCR: in case of positivity, bacteriologic culture was performed additionally.

Of the 73 reported cases of plague in Mongolia since 1998, 59% have been associated with close contact with infected marmots and 7% with eating raw marmot organs (data provided by National Centre for Zoonotic Diseases, Ministry of Health, Ulaanbaatar, Mongolia).

The rapid clinical course of the disease and the absence of signs of lymphadenitis with the detection of *Y. pestis* in the blood indicate that the husband died of septicaemic plague caused by the consumption of raw marmot infected with *Y. pestis*. *Y. pestis* infection occurred even though the man had received an EV76 vaccine one year previously. There is no evidence showing that the available *Y. pestis* vaccines provide humans with long-lasting immunity and protection from plague [8,9].

The combination of pharyngeal inflammation, swollen cervical lymph nodes, detection of the pathogen in cervical lymph node tissue and pharyngeal swab specimens, and the absence of fleabites indicate that the wife was also infected by consuming raw marmot meat. Pharyngeal inflammation and swollen cervical lymph nodes have previously been reported among patients who have consumed raw marmot meat [5,6,10]. The wifés concealment of consumption of raw marmot meat contributed to a delayed diagnosis. Because hunting marmots has been prohibited by the Mongolian government since 2014, Mongolians fear punishment when they admit this activity. Marmot, prepared as boodog (filled with hot stones), is a national dish in Mongolia (Figure 1(B–D)), and, because Mongolians assume that boodog has healthful benefits, marmot hunting is a covert activity. When marmots are prepared for boodog, especially before the fur is burnt away with a blowtorch, fleas infected with *Y. pestis* may change their host, thereby typically causing bubonic plague. *Marmota sibirica*, a species that also lives in Western Mongolia, exhibits a relatively high resistance to plague (50%–80% survive infection), a feature that has been suggested to play an important role in the persistence of the pathogen [3].

The habit of eating infected raw marmot meat is a possible means of infection with plague in Mongolia. Cooking efficiently inactivates *Y. pestis* [11]: thus, infections in Mongolia are associated with consumption of raw meat, not with the consumption of cooked boodog. The most important mode of *Y. pestis* infection in Mongolia is close contact with infected marmots. A study from Zambia [12] found that the risk of transfer of *Y. pestis* from infected fleas on captured animals to humans is higher during hunting and transporting of marmots rather than during preparation of animals for food. That study showed that hunting behaviour, mode of transportation of carcasses, and method of preparation of carcasses may substantially influence the risk of flea transmission.

A diagnosis of plague should be considered in areas with active plague foci, including the Bayan Ulgii aimag in the western part of Mongolia [3,13] where the dead couple resided. Only few reports of plague connected with the consumption of raw meat have been published to date; most cases are associated with the consumption of raw or undercooked camel meat [5–7,10]. The patients reported here had no fleabites and their predominant symptoms were bloody vomitus and sepsis in one case and gastrointestinal symptoms and pharyngeal inflammation in the other. Therefore, we conclude that the infections were caused by the consumption of raw marmot meat.

Marmot meat is considered a delicacy in Mongolia, but its consumption confers a risk of *Y. pestis* infection. In plague-endemic districts, healthcare professionals obtaining a patient´s medical history should ask about consumption of marmot and plague should be considered as a tentative diagnosis. Communicating the risk of *Y. pestis* infection after close contact with or consumption of marmot meat, especially raw meat, may increase the awareness of plague among the population.
